# Plasma Levels of Proprotein Convertase Subtilisin/Kexin Type 9 Are Inversely Associated with N-Terminal Pro B-Type Natriuretic Peptide in Older Men and Women

**DOI:** 10.3390/biomedicines10081961

**Published:** 2022-08-12

**Authors:** Francesco Spannella, Federico Giulietti, Roberta Galeazzi, Anna Passarelli, Serena Re, Chiara Di Pentima, Massimiliano Allevi, Paolo Magni, Riccardo Sarzani

**Affiliations:** 1 Internal Medicine and Geriatrics, IRCCS INRCA, 60129 Ancona, Italy; 2 Department of Clinical and Molecular Sciences, Università Politecnica delle Marche, 60126 Ancona, Italy; 3 Clinical Laboratory and Molecular Diagnostic, IRCCS INRCA, 60129 Ancona, Italy; 4 Department of Pharmacological and Biomolecular Sciences, Università degli Studi di Milano, 20133 Milan, Italy; 5 IRCCS MultiMedica, Sesto S. Giovanni, 20099 Milan, Italy

**Keywords:** PCSK9, NT-proBNP, older men and women, lipid metabolism, cardiovascular disease

## Abstract

Background and Aims: Cardiac natriuretic peptides (NPs) exert several metabolic effects, including some on lipid metabolism. Higher NPs levels are likely to be associated with a favorable lipid profile. In in vitro studies, NPs have been found to modulate low-density lipoprotein receptor (LDLR) trafficking by preventing proprotein convertase subtilisin/kexin type 9 (PCSK9) overexpression. The aim of our study is to investigate a possible association between plasma levels of PCSK9 and N-terminal pro B-type natriuretic peptide (NT-proBNP) in vivo. Methods: We performed a cross-sectional study on 160 consecutive older male and female patients hospitalized for medical conditions. Patients taking lipid-lowering drugs and patients with an admission diagnosis of acute heart failure were excluded. Fasting blood samples were collected after clinical stabilization of the acute illness, the day before discharge. Results: The mean age was 87.8 ± 6.4 years with a female prevalence (62.5%). The median NT-proBNP was 2340 (814–5397) pg/mL. The mean plasma PCSK9 was 275.2 ± 113.2 ng/mL. We found an inverse correlation between plasma PCSK9 and NT-proBNP (r = −0.280; *p* = 0.001). This association was confirmed after taking into account NT-proBNP tertiles (plasma PCSK9 levels: 317.4 ± 123.6 ng/mL in the first tertile, 283.3 ± 101.8 ng/mL in the second tertile, 231.3 ± 99.0 ng/mL in the third tertile, *p* = 0.001) and even after an adjustment for confounding factors (beta = −0.361, *p* = 0.001 for ln(NT-proBNP); beta = −0.330, *p* = 0.001 for NT-proBNP tertiles). The strength of the correlation between plasma PCSK9 and NT-proBNP was likely greater in patients affected by type 2 diabetes mellitus (r = −0.483; *p* = 0.006) and in male patients (r = −0.431, *p* = 0.001). Conclusion: The inverse association found between PCSK9 and NT-proBNP plasma levels in our real-life clinical study supports the hypothesis that NPs may play a role in cholesterol metabolism, possibly through an inhibitory action on circulating PCSK9 concentrations, thus increasing the availability of LDLR.

## 1. Introduction

Dyslipidemia and arterial hypertension are the major modifiable cardiovascular (CV) risk factors for CV morbidity and mortality. Low-density lipoproteins (LDLs) and the cholesterol bound to them (LDL-C) are currently considered a cause of atherosclerosis [1]. Therefore, understanding the mechanisms that regulate circulating LDL-C levels is crucial.

Proprotein convertase subtilisin/kexin type 9 (PCSK9), a member of the proprotein convertase family, is highly expressed in the liver and to a lesser extent in other tissues, such as the intestine, kidney, aorta and adipose tissue [2,3,4]. PCSK9 plays a critical role in the regulation of cholesterol homeostasis, controlling the recycling of LDL receptors (LDLR). This protein binds to and degrades the LDLR in hepatocytes by increasing endosomal and lysosomal degradation and thus contributing to high-circulating LDL-C [5,6]. PCSK9, mainly secreted by hepatocytes, circulates in humans in both an intact form and a major truncated form with a significant proportion (20–40%) bound to lipoproteins [7,8]. Cardiac natriuretic peptides (NPs) play a relevant role not only in blood pressure regulation, blood volume and sodium balance, but also in lipid metabolism by stimulating lipolysis, increasing energy expenditure, enhancing lipid oxidation, browning white adipocytes, and decreasing inflammatory cytokines and insulin resistance [9,10,11]. This group of CV hormones that includes A-type NPs and B-type NPs is released by the heart as a result of muscular wall stretch due to increased intraventricular volume and/or cardiac transmural pressure [9,10,11]. Both of the active forms of these two CV hormones (ANP and BNP) act through the activation of a guanylyl cyclase-coupled transmembrane receptor (NPRA), expressed mainly in vascular smooth muscle and endothelial cells, adipose tissue, kidneys, adrenal gland, liver and brain, and to a lesser extent, in the heart and skeletal muscle [9,10,11]. BNP and its N-terminal non-active prohormone (NT-proBNP), which is more stable and has a longer half-life, are widely used in common clinical practice for the diagnosis and management of heart failure (HF) and for the prognostic stratification of patients with a high CV risk [12]. Clinical studies have found an association between higher NPs levels and a favorable lipid profile, with an inverse association between circulating NT-proBNP and plasma LDL-C, even for a wide range of NT-proBNP levels [10,13]. Moreover, we recently found a direct mechanistic relationship between ANP and PCSK9 expression. In our recent experimental study, ANP appeared to reduce the expression of PCSK9 in human adipocytes, previously stimulated by insulin and LDL [4]. This previous experimental study laid the foundation for the design of the present clinical study.

Based on this knowledge, we aim to investigate whether there is an association between plasma PCSK9 and NPs (NT-proBNP) in a real-life clinical setting of an older population of hospitalized patients who commonly show increased NT-proBNP levels.

## 2. Materials and Methods

### 2.1. Study Population

We performed a cross-sectional study on 160 older male and female adults admitted to our Internal Medicine and Geriatrics Department from January 2019 to December 2020. The following exclusion criteria were applied: current therapy with lipid-lowering drugs or other drugs that could have affected lipid profile (i.e., corticosteroids), clinical conditions that could have affected lipid profile (such as hypo and hyperthyroidism, end-stage renal or liver diseases, advanced cancer and cachexia) or an admission diagnosis of acute HF confirmed by a cardiologist (after clinical examination and echocardiography in the emergency room). Given the aim of our clinical study, the sample taken into account allowed us to investigate wide ranges of NT-proBNP due to the high prevalence of underlying heart disease in older populations [13]. Clinical investigations were conducted according to the principles expressed in the Declaration of Helsinki. This observational study was approved by the local institutional ethics committee (CE INRCA, Ancona, Italy, protocol code 104/DGEN, 5 April 2019).

### 2.2. Clinical and Lab Parameters

Medical history was evaluated for each enrolled patient. Fasting blood samples were collected after clinical stabilization of the acute illness, the day before discharge. We took into account the following laboratory parameters: NT-proBNP, hemoglobin (Hgb), estimated glomerular filtration rate (eGFR), glycaemia, albumin, total cholesterol (TC), high-density lipoprotein cholesterol (HDL-C), triglycerides and calculated low-density lipoprotein cholesterol (LDL-C). The GFR was estimated using the Chronic Kidney Disease Epidemiology Collaboration (CKD-EPI) creatinine equation. The LDL-C was calculated using a modified Friedewald method proposed by Martin et al. [14]. Furthermore, we took into account Non-HDL-C that was calculated by subtracting HDL-C from TC, as a measure of the total cholesterol carried by all the atherogenic ApoB-containing lipoproteins, and (Non-HDL-C + Non-LDL-C) that was calculated by subtracting (HDL-C + LDL-C) from TC, as an estimate of the very low-density lipoprotein cholesterol (VLDL-C) and the cholesterol carried by fasting remnant lipoproteins.

As previously reported [13], to evaluate patients’ functional status, the 7-point MDS Activities of Daily Living (ADL) hierarchy scale was used. The ADL hierarchy scale groups activities of daily living according to the stage of the disablement process in which they occur [15]. The ADL hierarchy scale ranges from 0 (no dependence) to 6 (total dependence). The ADL disability was categorized as follows: no impairment (ADL hierarchy scale score < 2), assistance required (ADL hierarchy scale score 2–4), and dependence (ADL hierarchy scale score ≥ 5). Cognitive impairment was based on a previous documented diagnosis, given that the result of any cognitive test could have been altered by the acute phase. The Geriatric Index of Comorbidity (GIC) was used to determine the burden of comorbidities and it was categorized as low comorbidity (GIC classes 1 or 2) and high comorbidity (GIC classes 3 or 4) [16].

### 2.3. NT-proBNP Assay

After blood sampling, the NT-proBNP was measured using an Elecsys proBNPII electrochemiluminescence immunoassay in a Cobas e601 immunoassay Roche analyzer (Roche Diagnostics S.p.A, Monza, Italy). This assay contains two monoclonal antibodies that recognize epitopes located in the N-terminal part (1–76) of the proBNP (1–108) [13].

### 2.4. PCSK9 Assay

The blood samples for PCSK9 assay were stored at −80° C and PCSK9 concentrations were measured using a commercial ELISA kit (R&D Systems, Minneapolis, MN, USA) which was able to recognize free and LDLR-bound PCSK9 [17]. The plasma samples were diluted at 1:20 and incubated onto a microplate pre-coated with a monoclonal antibody specific for human PCSK9. The sample concentrations were obtained by generating a four-parameter logistic curve fit. The minimum detectable PCSK9 concentration was 0.219 ng/mL. The intra- and inter-assay coefficients of variability were 3.8% and 5.8%, respectively.

### 2.5. Statistical Analysis

The data were analyzed with the Statistical Package for Social Science version 21 (SPSS Inc., Chicago, IL, USA). A value of *p* < 0.05 was defined as statistically significant. The continuous variables were checked for normality and are expressed as mean ± standard deviation or as median and interquartile ranges for the variables markedly skewed. The categorical variables are expressed as a percentage. PCSK9 and NT-proBNP were both analyzed as continuous and discrete variables. The NT-proBNP tertiles: first tertile ≤ 1203 pg/mL; second tertile 1204–3814 pg/mL; third tertile ≥ 3815 pg/mL. The PCSK9 tertiles: first tertile < 216.8 ng/mL; second tertile 216.8–316.5 ng/mL; third tertile ≥ 316.6 ng/mL. The NT-proBNP was naturally logarithmically transformed to normalize its distributions. The χ^2^ test was used to analyze the differences between categorical variables. Pearson and Spearman correlations were used to analyze the relationship between continuous variables. The analysis of variance (ANOVA) and Kruskal–Wallis test were used to compare quantitative variables. In addition to age and sex, variables with significant associations identified on univariate analyses were included in multiple linear regression analyses to create adjusted models.

## 3. Results

### 3.1. General Characteristics

General characteristics of the entire study population and according to PCSK9 tertiles are described in Table 1. Mean age was 87.8 ± 6.4 years with female prevalence (62.5%). Median length of stay was 9 (6–14) days. The study population showed a high burden of comorbidity, in particular, history of hypertension, chronic HF and cognitive impairment were the most prevalent conditions. There were no significant differences among PCSK9 tertiles regarding clinical characteristics, except for history of coronary artery disease (CAD), which was more prevalent in the second PCSK9 tertile. Mean albumin levels tended to drop, while median triglyceride levels increased significantly, with increasing PCSK9 tertiles (Table 1). Patients with higher NT-proBNP levels were older and had a high burden of comorbidity, and especially a higher prevalence of chronic HF and lower eGFR (Supplementary Table S1).

### 3.2. Plasma PCSK9 and NT-proBNP

A trend of increasing LDL-C levels across PCSK9 tertiles was found, albeit in the absence of statistical significance, while (Non-HDL-C + Non-LDL-C) significantly increased across PCSK9 tertiles (Table 1). Inversely, LDL-C levels tended to decrease with increasing NT-proBNP tertiles (Supplementary Table S1). We found significant direct correlations between LDL-C and plasma PCSK9 (r = 0.180; *p* = 0.031), and between (Non-HDL-C + Non-LDL-C) and plasma PCSK9 (r = 0.326; *p* < 0.001), while we found a significant inverse correlation between LDL-C and NT-proBNP (r = −0.193; *p* = 0.026).

**Figure 1:**
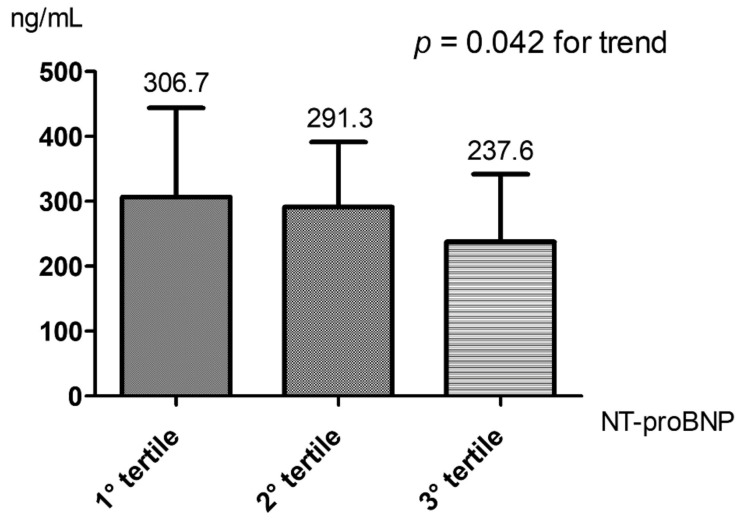
PCSK9 levels according to NT-proBNP tertiles in the overall population.

There was a decrease in PCSK9 levels with increasing NT-proBNP levels, showing a significant inverse correlation (r = −0.280; *p* = 0.001). The strength of the correlation between plasma PCSK9 and NT-proBNP was likely greater in patients affected by type 2 diabetes mellitus (T2DM) (r = −0.483; *p* = 0.006) compared to non-diabetic patients (r = −0.235; *p* = 0.012). In a sub-analysis according to sex, the significant correlation was confirmed only in the males (r = −0.431, *p* = 0.001 for males and r = −0.191, *p* = 0.066 for females). The significant trend of PCSK9 levels according to NT-proBNP tertiles was present in the overall population (Figure 1) and in the sub-analysis according to sex (Supplementary Figure S1). The inverse association between PCSK9 and NT-proBNP levels remained significant even after statistical adjustments (Table 2 and Table 3).

## 4. Discussion

In the present real-life clinical study, we found an inverse association between plasma NT-proBNP and PCSK9 concentrations in an older population hospitalized for medical conditions characterized by a wide range of plasma NT-proBNP levels, mostly due to increased ventricular wall stress/overload. The strength of the association was likely greater in patients affected by type 2 diabetes mellitus and in male patients, suggesting a possible gender difference. Lower levels of circulating PCSK9 are expected to increase the expression of LDLR on hepatocyte surfaces, leading to reduced circulating LDL-C levels [18]. Accordingly, we found that LDL-C levels were directly associated with plasma PCSK9, but inversely associated with NT-proBNP in our study, in agreement with previous findings observed in middle-aged patients [10].

The major determinants of PCSK9 levels and their impact on the onset of CV risk factors, vascular damage and CV events are still debated, given the disagreement of several previous studies on these topics [19,20,21,22,23,24,25,26]. Our results expand the knowledge on the metabolic role of cardiac NPs in the regulation of lipid metabolism.

In this study, we chose to take into account NT-proBNP, given its established use in daily clinical practice as a key diagnostic and prognostic marker of heart diseases [12], and its association with LDL-C levels that has already been found in an older population with similar characteristics. In fact, we found a significant inverse association between NT-proBNP and LDL-C, even after adjusting for several confounding factors, in a previous study on 288 oldest-old patients [13]. In the present study, we found a weak positive association between circulating PCSK9 and LDL-C, in agreement with previous studies on untreated patients [26], although it is known that plasma PCSK9 levels explain less than 8% of the variation in LDL-C levels [27]. A positive association between plasma PCSK9 levels and (Non-HDL-C + Non-LDL-C), used as an estimate of the VLDL-C and the cholesterol carried by fasting remnant lipoproteins, was also found in our study. Based on these findings, it would be interesting to investigate the relationship between PCSK9, NPs, and the lipid composition of circulating lipoproteins in future studies.

Several studies found higher circulating PCSK9 levels in adult patients with metabolic syndrome, T2DM, and obesity [20,21,22]. At the same time, these subjects have overexpressed NPs clearance receptor (NPRC) in their adipocytes, resulting in lower NPs circulating levels [10]. Furthermore, treatment with ANP in human cultured adipocytes reduced insulin-induced PCSK9 expression, especially in the context of hyperglycemia, simulating a clinical condition of insulin resistance/T2DM in our recent experimental study [4]. Based on these preclinical data, we performed a sub-analysis in patients with T2DM, finding an even stronger inverse association between PCSK9 and NT-proBNP in these patients. At the same time, given the aforementioned in vitro data on human cultured adipocytes [4], it is plausible that a possible in vivo association with PCSK9 also exists for ANP, a cardiac hormone exerting more physiological actions and less affected by pathological cardiac stress [11].

NT-proBNP, more frequently assayed in the clinical routine for the diagnosis and management of heart failure, due to its greater stability and longer half-life, is closely associated and considered a secretion marker for BNP, the active peptide. Both ANP and BNP recognize the same ligand in NPRA, inducing an intracellular generation of the second messenger cyclic guanosine monophosphate (cGMP), which mediates NPs’ biological effects. Therefore, similar cardiovascular and metabolic actions are expected from these two peptides [11]. Further studies are needed to better clarify this aspect. Although the design of the present study does not allow a cause–effect link to be established, the experimental data reported above allow us to hypothesize that the association found between NT-proBNP and PCSK9 could be due to the possible downregulation of PCSK9 expression exerted by NPs. This is likely present not only in the adipose tissue [4], but also in the liver, given that circulating PCSK9 is mainly secreted by hepatocytes [28]. This hypothesis paves the way for further ad hoc experimental studies in order to better understand how cardiac-born NPs affect lipid metabolism.

Higher NPs levels are associated with a more favorable lipid profile, in particular, with lower LDL-C and triglycerides, and higher HDL-C. Several mechanisms behind NPs’ influence on lipid metabolism are already known [10]. NPs receptors are expressed in several organs and tissues, such as vessels, kidney, skeletal muscle and adipose tissue [9,10,11]. NPs promote thermogenesis, the use of triglycerides derived from plasma triglyceride-rich lipoproteins, and lipolysis. Therefore, NPs induce higher free fatty acid availability as a substrate for oxidation in tissues, such as skeletal muscle, liver and adipose tissue [9,29,30].

To the best of our knowledge, this is the first study that was specifically designed to investigate the association between PCSK9 and NPs circulating levels. Several previous studies on younger populations collaterally assessed the reciprocal trend of PCSK9 and NT-proBNP, mainly in descriptive analyses, without performing statistical adjustments, thus finding conflicting results [25,31,32,33,34]. Moreover, all of these studies took into account blood samples collected in acute cardiovascular conditions, such as acute coronary syndrome or a worsening of HF that could have affected the circulating levels of both NPs and PCSK9. Therefore, given these relevant potential biases, these studies were not able to adequately investigate the association that was the main scope of our study. They also showed much lower NT-proBNP levels compared to those found in our study [25,31,32,33,34]. Therefore, the results of the present study, that suggest an inverse relationship between PCSK9 and NT-proBNP in an older cohort, cannot be easily compared to such different previous studies and appear novel and peculiar to this population [19].

Older patients tend to have higher NT-proBNP levels than younger patients, mainly due to the presence of underlying heart diseases [13]. This condition allowed us to investigate the possible association between PCSK9 and a wide range of NT-proBNP levels. In this study, we minimized the possibility of confounding factors through the careful selection of the study population and a multivariate analysis, in order to exclude potential confounding factors associated with older age. The main covariates, commonly associated in the literature with older individuals, and therefore representing potential significant confounding factors in this peculiar population, such as albumin, cognitive impairment, and burden of comorbidity, were included in the adjustment models, and were found to not affect the results.

However, the relationship found in the present study could also be dependent on factors that still need to be investigated, given the complex interaction between lipid metabolism, PCSK9 and the heart [19]. Indeed, a recently published study on PCSK9 knock-out mice found that PCSK9 deficiency could negatively affect cardiac lipid metabolism (increased left ventricular thickness and an increased cardiac accumulation of lipid droplets were associated with a reduced density of mitochondrial cristae leading to impaired oxidative phosphorylation and mitochondrial metabolism) in a LDLR-independent manner, contributing to the development of HF and secondarily to high NPs levels [35].

### Study Limitations

Our study also has limitations that need to be pointed out. First, our investigation is a cross-sectional study that did not allow us to establish causality for the associations observed, and we could only speculate regarding the possible underlying biological mechanisms. Moreover, our findings in an older population may not be extended to younger populations. Finally, in order to minimize the possibility of confounding factors, we carefully excluded patients taking drugs and patients affected by conditions that could have interfered with the main associations analyzed. This, at least in part, contributed to a decrease in the sample size of the study. However, retrospective power calculation showed that our study was able to find the observed correlation between NT-proBNP and PCSK9 (Βeta = −0.361) with a power of 88% (α-error of 0.05), thus confirming the adequacy of the sample size analyzed.

## 5. Conclusions

Dyslipidemia is one of the most common CV risk factors, and a major determinant of both CV disease and mortality [36]. The inverse association found between PCSK9 and NT-proBNP in our real-life clinical study supports the hypothesis that NPs play a role in cholesterol metabolism, also through a probable inhibitory action on PCSK9, thus increasing the LDLR availability. Our clinical findings, together with previous laboratory evidence, pave the way for further preclinical and clinical studies aimed at investigating how NPs may affect PCSK9 and therefore circulating cholesterol levels. These findings, if confirmed by further experimental and clinical studies, could have a direct clinical impact on both the prevention and management of CV diseases, also given that several new therapeutic agents capable of stimulating NPs systems are in development and under experimentation.

## Figures and Tables

**Table 1:** General characteristics of the entire study population according to PCSK9 tertiles.

*Clinical Characteristics*	All Patients (n° 160)	1st PCSK9 Tertile (n° 54)	2nd PCSK9 Tertile (n° 53)	3rd PCSK9 Tertile (n° 53)	*p*
Age (years)	87.8 ± 6.4	88.2 ± 5.8	87.2 ± 6.7	88.0 ± 6.5	0.690
Sex (female)	62.5%	64.8%	60.4%	62.3%	0.893
GIC (high comorbidity)	70.3%	78.0%	69.8%	63.5%	0.274
ADL hierarchy scale: assistance required	54.8%	58.0%	54.7%	51.9%	0.471
ADL hierarchy scale: dependence	12.9%	8.0%	18.9%	11.5%	
History of hypertension	69.7%	76.0%	73.6%	59.6%	0.148
Type 2 diabetes mellitus	21.9%	24.0%	24.5%	17.3%	0.612
History of CAD	12.3%	8.0%	22.6%	5.8%	0.017
History of chronic HF	38.1%	40.0%	45.3%	28.8%	0.210
Previous TIA/stroke	15.5%	18.0%	13.2%	15.4%	0.798
Cognitive impairment	45.7%	57.1%	36.0%	44.2%	0.104
*Lab parameters*					
TC (mg/dL)	151.2 ± 39.2	147.4 ± 45.0	148.5 ± 35.0	159.0 ± 35.9	0.297
HDL-C (mg/dL)	46.2 ± 16.9	49.3 ± 17.8	43.9 ± 14.9	45.0 ± 17.7	0.240
LDL-C (mg/dL)	82.7 ± 31.0	79.1 ± 35.5	80.9 ± 26.2	89.1 ± 29.7	0.256
Non-HDL-C (mg/dL)	102.4 ± 36.5	98.1 ± 40.2	104.6 ± 32.8	104.6 ± 36.2	0.586
(Non-HDL-C + Non-LDL-C)	20.0 (15.0–25.0)	16.0 (13.0–22.0)	21.0 (15.0–28.0)	22.0 (17.0–29.0)	0.026
Triglycerides (mg/dL)	100.0 (73.3–122.8)	78.0 (64.3–110.8)	104.0 (78.0–140.0)	112.0 (86.0–145.0)	<0.001
NT-proBNP (pg/mL)	2340 (814–5397)	3896 (1234–6776)	2970 (1124–6346)	1431 (510–3341)	0.005
Plasma PCSK9 (ng/mL)	275.2 ± 113.2	157.5 ± 42.2	262.5 ± 27.4	407.8 ± 64.1	<0.001
Hgb (g/dL)	11.4 ± 1.8	11.1 ± 1.8	11.4 ± 1.7	11.6 ± 2.0	0.295
eGFR (mL/min/1.73 m^2^)	54.1 ± 24.6	52.8 ± 23.1	55.6 ± 24.2	53.9 ± 26.7	0.846
Glycaemia (mg/dL)	102.0 (86.5–137.5)	95.5 (83.0–127.0)	103.0 (87.0–137.0)	109.0 (84.0–140.8)	0.457
Albumin (g/dL)	3.3 ± 0.6	3.4 ± 0.5	3.4 ± 0.6	3.2 ± 0.5	0.053

GIC: Geriatric Index of Comorbidity; ADL: activities of daily living; CAD: coronary artery disease; HF: heart failure; TIA: transient ischemic attack; TC: total cholesterol; HDL-C: high-density lipoprotein cholesterol; LDL-C: low-density lipoprotein cholesterol; NT-proBNP: N-terminal pro B-type natriuretic peptide; PCSK9: proprotein convertase subtilisin/kexin type 9; Hgb: hemoglobin; eGFR: estimated glomerular filtration rate.

**Table 2:** Linear regression analysis for association between plasma PCSK9 (dependent variable) and ln(NT-proBNP).

	Βeta	B (95% CI)	*p*
ln(NT-proBNP)	−0.361	−31.33 (−49.49–−13.16)	0.001
Age (years)	0.070	1.37 (−2.30–5.04)	0.461
Sex (ref. female)	−0.051	−12.21 (−51.50–27.08)	0.540
Albumin (g/dL)	−0.232	−47.23 (−83.93–10.54)	0.012
History of chronic HF	0.065	15.02 (−29.76–59.80)	0.508
Cognitive impairment	−0.012	−2.80 (−42.80–37.20)	0.890
GIC (ref. low comorbidity)	−0.107	−26.87 (−70.00–16.27)	0.220

B = unstandardized regression coefficient (represents the amount of change in plasma PCSK9 levels for a one-unit increase in dependent variable); beta = standardized regression coefficient (indicates the influence of independent variables on dependent variables). NT-proBNP: N-terminal pro B-type natriuretic peptide; HF: heart failure; GIC: Geriatric Index of Comorbidity.

**Table 3:** Linear regression analysis for association between plasma PCSK9 (dependent variable) and NT-proBNP tertiles.

	Βeta	B (95% CI)	*p*
NT-proBNP tertiles	−0.330	−45.96 (−73.21–−18.70)	0.001
Age (years)	0.048	0.95 (−2.65–4.54)	0.604
Sex (ref. female)	−0.065	−15.52 (−55.18–24.15)	0.440
Albumin (g/dL)	−0.210	−42.85 (−79.44–−6.27)	0.022
History of chronic HF	0.033	7.71 (−35.60–51.03)	0.725
Cognitive impairment	−0.029	−6.58 (−46.51–33.35)	0.745
GIC (ref. low comorbidity)	−0.112	−28.02 (−71.17–15.13)	0.201

B = unstandardized regression coefficient (represents the amount of change in plasma PCSK9 levels for a one unit increase in dependent variable); beta = standardized regression coefficient (indicates the influence of independent variables on dependent variables). NT-proBNP: N-terminal pro B-type natriuretic peptide; HF: heart failure; GIC: geriatric index of comorbidity.

## Data Availability

The data presented in this study are available on request from the corresponding author.

## Supplementary Materials

The following supporting information can be downloaded at: www.mdpi.com/article/10.3390/biomedicines10081961/s1, Table S1: General characteristics according to NT-proBNP tertiles.; Figure S1: PCSK9 levels according to NT-proBNP tertiles in males (Panel A) and females (Panel B).
