# Supplementary material for: Plasma Levels of Proprotein Convertase Subtilisin/Kexin Type 9 Are Inversely Associated with N-Terminal Pro B-Type Natriuretic Peptide in Older Men and Women
Source: Biomedicines. 2022 Aug 12;10(8):1961. doi: 10.3390/biomedicines10081961 (PMC9405766; doi:10.3390/biomedicines10081961)
Supplement: Supplementary file 1 [file biomedicines-10-01961-s001.zip › biomedicines-1804619-supplementary.pdf]

biomedicines

MDPI

Supplementary Materials

Table S1. General characteristics according to NT-proBNP tertiles.

Clinical characteristics	**1st NT-proBNP tertile (n° 54)**	**2nd NT-proBNP tertile (n° 53)**	**3rd NT-proBNP tertile (n° 53)**	***p***
Age (years)	85.0 ± 6.5	89.2 ± 4.3	90.6 ± 4.6	<0.001
Sex (female)	54.0%	64.0%	71.4%	0.196
GIC (high comorbidity)	59.2%	75.5%	80.9%	0.048
ADL Hierarchy Scale: Assistance required	42.9%	55.1%	68.1%	0.077
ADL Hierarchy Scale: Dependence	20.4%	12.2%	4.3%	
History of Hypertension	67.3%	66.7%	79.2%	0.315
Type 2 Diabetes Mellitus	20.4%	22.9%	20.8%	0.950
History of CAD	8.2%	16.7%	14.6%	0.432
History of chronic HF	18.4%	37.5%	64.6%	<0.001
Previous TIA/Stroke	12.2%	16.7%	18.8%	0.669
Cognitive impairment	32.7%	56.3%	50.0%	0.054
Lab parameters				
TC (mg/dL)	161.1 ± 38.8	150.1 ± 40.9	143.0 ± 34.2	0.090
HDL-C (mg/dL)	46.9 ± 17.9	45.8 ± 17.0	43.9 ± 14.6	0.699
LDL-C (mg/dL)	91.2 ± 29.8	81.0 ± 31.2	77.2 ± 30.0	0.095
Non-HDL-C (mg/dl)	104.7 ± 37.2	98.7 ± 34.3	104.3 ± 38.5	0.674
(Non-HDL-C + Non-LDL-C)	21.5 (16.3-23.0)	20.0 (14.0-27.5)	19.0 (15.0-28.0)	0.415
Triglycerides (mg/dL)	107.5 (83.0-117.0)	98.5 (70.8-137.5)	96.0 (73.0-137.0)	0.666
NT-proBNP (pg/mL)	491 (353-821)	2434 (1748-3311)	7552 (5397-12267)	<0.001
Hgb (g/dL)	11.6 ± 1.7	11.2 ± 2.2	11.3 ± 1.5	0.526
eGFR (ml/min/1.73 m²)	62.4 ± 24.4	55.8 ± 22.6	40.6 ± 20.8	<0.001
Glycaemia (mg/dL)	112.0 (89.0-140.5)	102.0 (85.0-135.5)	101.0 (81.5-141.5)	0.480
Albumin (g/dL)	3.4 ± 0.5	3.2 ± 0.6	3.3 ± 0.6	0.158

GIC: Geriatric Index of Comorbidity; ADL: Activities of Daily Living; CAD: Coronary Artery Disease; HF: Heart Failure; TIA: Transient Ischemic Attack; TC: Total Cholesterol; HDL-C: High-Density Lipoprotein Cholesterol; LDL-C: Low-Density Lipoprotein Cholesterol; NT-proBNP: N-terminal pro B-Type Natriuretic Peptide; Hgb: Hemoglobin; eGFR: estimated Glomerular Filtration Rate.

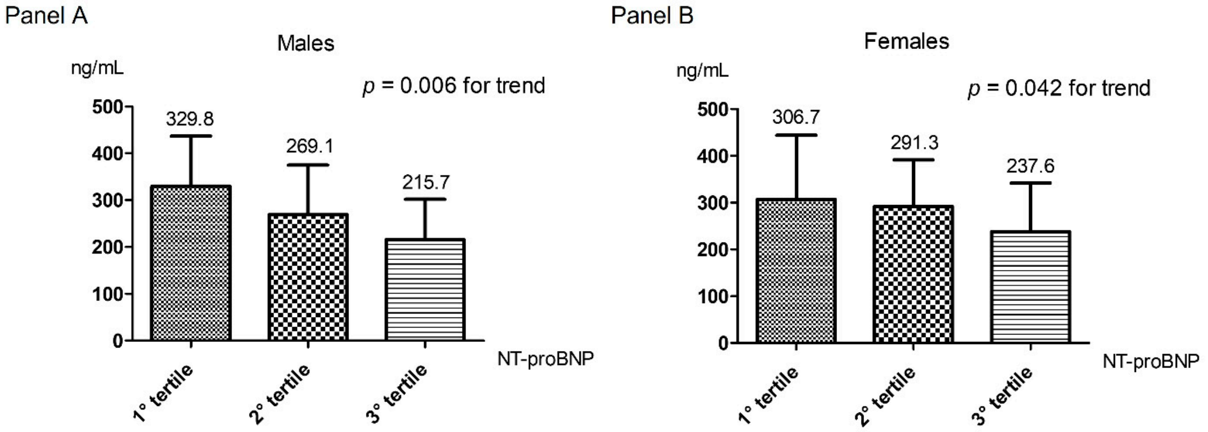

Figure S1. PCSK9 levels according to NT-proBNP tertiles in males (Panel A) and females (Panel B).
